# Cardiovascular magnetic resonance of quinticuspid aortic valve with aortic regurgitation and dilated ascending aorta

**DOI:** 10.1186/1532-429X-11-28

**Published:** 2009-08-11

**Authors:** Yanfeng Meng, Lijun Zhang, Zhaoqi Zhang, Yongmei Wang, Xiaoming Yang

**Affiliations:** 1 Radiology, Beijing Anzhen Hospital, Capital Medical University, Beijing 100029, PR China; 2 Image-Guided Bio-Molecular Interventions Research, Radiology, University of Washington, Seattle, WA 98109, USA

## Abstract

We report a rare case of a quinticuspid aortic valve associated with regurgitation and dilation of the ascending aorta, which was diagnosed and post-surgically followed up by cardiovascular magnetic resonance and dual source computed tomography.

## Background

Aortic valve malformation is a common congenital cardiac anomaly in the adult population. Dysfunction of the malformed aortic valve has been shown to cause significant morbidity and mortality. Among the malformations, bicuspid is the most common form, which has been identified in 1–2% of population with male predominance [1]. To our knowledge, an aortic valve containing 5 leaflets (quinticuspid) is very rare [2]. Here we report a case of quinticuspid aortic valve associated with aortic regurgitation and dilation of the ascending aorta.

## Case report

**Figure 1:**
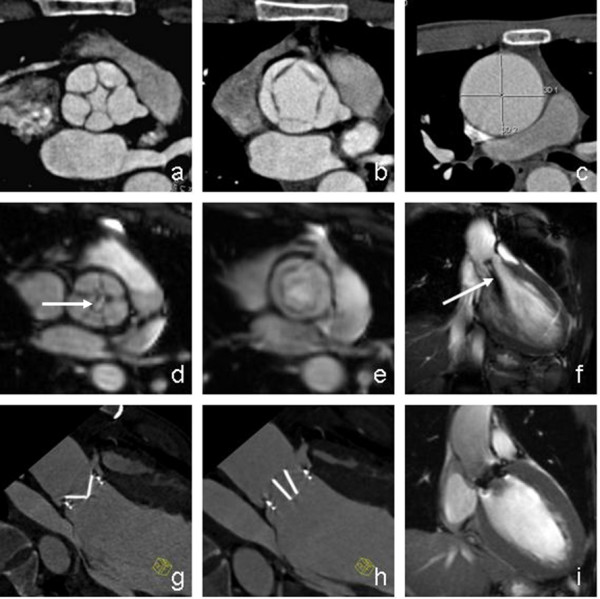
**CT and CMR of quinticuspid aortic valve pre- and post-operative examination**. (a) Dual source CT revealed malformed aortic valve in diastolic phase. (b) Dual-source CT showed malformed aortic valve in systolic phase. (c) Dilated ascending aorta. (d) CMR revealed malformed aortic valve in diastolic phase. (e) CMR showed malformed aortic valve in systolic phase. (f) Regurgitation in diastolic phase revealed as signal void (arrow). (g) Dual source CT showed mechanical valve in systolic phase. (h) Dual source CT showed mechanical valve in diastolic phase. (i) There is no regurgitation in diastolic phase.

A 24-year-old Chinese man presented with worsening symptoms of palpitation, chest discomfort and shortness of breath over a two-year period. There was no prior history of rheumatic fever or endocarditis. On physical examination, his pulse was 68 per minute with a blood pressure of 145/41 mmHg. Subsequently, the patient underwent a series of imaging studies including echocardiography, chest X-ray, dual source x-ray computed tomography (CT) and cardiovascular magnetic resonance (CMR) (Figure 1), which showed a quinticuspid aortic valve. The CT scan demonstrated an aortic valve with 5 valve cusps and slightly thickened leaflet edges. In the systolic phase, the valve orifice measures 6.3 cm^2 ^in area and opened like a pentagon, while in the diastolic phase, the valve orifice measured 1.2 cm^2 ^in area and closed like pentagram (Figure 1a, b, d, e). Five sinuses of valsalva were identified with variable sizes, of which the posterior sinus was notably smaller than all other sinuses. CMR SSFP for the cine sequence demonstrated incomplete closure of the five aortic valve cusps, in which a small orifice was found in diastolic phase (Figure 1d, Additional file 1). In addition, aortic regurgitation was noted as blood back-flow was demonstrated from aorta to left ventricle at diastolic phase (Figure 1f). Phase contrast images demonstrated the peak flow velocity of regurgitation at 480 cm/s. Furthermore, the patient was found to have an enlarged left ventricle chamber (484 ml for the end diastolic volume with a normal range of 77–195 ml, and 270 ml for the end systolic volume with a normal range of 19–72 ml), and a dilated ascending aorta (49 mm in diameter; normal range: < 40 mm) (Figure 1c, f).

Given the aggravating symptoms of aortic insufficiency and imaging finding of aortic valve anomaly, the patient proceeded to receive an aortic valve replacement with a concurrent Robicsek procedure, a surgery of reinforced aortoplasty. The aortic root was transected and the malformed aortic valve was replaced with an ON-X25 mechanical valve (ON-X technologies, Inc., Austin, Texas, USA). A 32 mm artificial vessel was used to enwrap the aortic root with Robicsek procedure. Postoperative CT and CMR scans were performed and showed functional artificial aortic valve with no regurgitations detected (Figure 1g–i).

## Discussion

In congenital aortic anomalies, the number of aortic leaflets can deviate from the normal three leaflets. Bicuspid is the most common form. Uni- and quadricuspids are seen much less frequently [3-6]. To our knowledge, this is the first reported case of quinticuspid aortic valve accompanied with detailed imaging studies.

The cause of this type of aortic valve anomaly is unknown. During embryologic development, the aortic valve normally develops into three commissures and three leaflets. If this process fails, the valve may be less than three cusps. If more than three commissures develop, it would cause a valve with four or more cusps. Valves with abnormal numbers may open improperly to cause stenosis, or may close incompletely resulting in regurgitation. Patients with these abnormalities may expect to develop hemodynamically significant complications at some point in their lifetime. In our case, the patient developed left ventricular chamber dilation and ascending aorta dilation at a relatively young age of 24 years.

CT and CMR have been demonstrated to be effective imaging methods for accurate diagnosis of aortic valve malformation [7-9]. In this case, accurate preoperative diagnosis of aortic valve abnormality was made easier with dual source CT and CMR. CT yielded images with higher spatial and temporal resolution than CMR. We were able to demonstrate detailed anatomy of the quinticuspid aortic valve and size of each sinus of valsalva. However, CMR yielded hemodynamic data in addition, including a detailed assessment of aortic regurgitation with signal changes from the abnormal blood flow. Both CT and MR are useful in postoperative follow-up of the morphology and function of the artificial valve. Although echocardiography is both sensitive and specific in detecting bicuspid aortic valves, it proved to be less useful in this case due to poor echocardiographic windows, and did not identify the 5 closely related leaflets.

In general, patients with known aortic valve anomaly are closely followed up to monitor the symptoms and signs relating to aortic stenosis or insufficiency. The goal is to prevent permanent damage to the left ventricle, or excessive dilation of the ascending aorta leading to aortic dissection and aneurysm. Same cases may necessitate surgical intervention, which commonly include aortic valve replacement with procedures to manage dilated ascending aorta. Given the fact that the currently available valve substitutes are not ideal for young patients, some choose valve repair instead. Tricuspidization is suitable for bicuspid and quadricuspid valves [10-12], and bicuspidization, for unicuspid aortic valves [13]. Our patient was successfully managed with aortic valve replacement with a Robicsek procedure for aortic root narrowing.

## Conclusion

In summary, we have illustrated an unusual case of quinticuspid aortic valve in a young adult presented with aortic regurgitation, left ventricular enlargement, and dilation of the ascending aorta. This case also demonstrates the value of CT and CMR in the diagnosis of this condition.

## Consent

Written informed consent was obtained from the patient for publication of this case report and any accompanying images. A copy of the written consent is available for review by the Editor-in-Chief of this journal.

## Competing interests

The authors declare that they have no competing interests.

## Authors' contributions

YM was responsible for the analysis and interpretation of the images, drafting of the manuscript. LZ was responsible for the generation of images, analysis and reconstruction of images. ZZ was responsible for the revision of the manuscript. YW was responsible for acquirement of images and provision of clinical information. XY was responsible for the revision and editing of the manuscript

## Authors' information

YM, MD, Fellow of Radiology. LZ, MD, Resident of Radiology. ZZ, MD, Professor and Chairman of Radiology. YW, MD, Associated professor of Radiology. XY, MD, PhD, Professor and Director of Image-Guided Bio-Molecular Interventions Research, Radiology

## Supplementary Material

Additional file 1 **CMR cine of aortic valve movement**. the CMR movie showed the movement of the quinticuspid aortic valve in a cardiac cycle. Click here for file
